# Predictors of Relapse after Inpatient Opioid Detoxification during 1-Year Follow-Up

**DOI:** 10.1155/2016/7620860

**Published:** 2016-09-18

**Authors:** Harsh Chalana, Tanu Kundal, Varun Gupta, Amandeep Singh Malhari

**Affiliations:** Department of Psychiatry, Sri Guru Ram Dass Institute of Medical Sciences and Research, Amritsar 143501, India

## Abstract

*Introduction*. Relapse rate after opioid detoxification is very high. We studied the possibility that predetoxification patient characteristics might predict relapse at follow-up and thus conducted this 1-year follow-up study to assess the predictors of relapse after inpatient opioid detoxification.* Materials and Methods*. We conducted this study in our tertiary care institute in India over two-year time period (1 Jan 2014 to 31 Dec 2015). Out of 581 patients admitted, 466 patients were considered for study.* Results and Discussion*. No significant difference was found between relapsed and nonrelapsed patients regarding sociodemographic profile; however substance abuse pattern and forensic history showed significant differences. Relapsed patients abused greater amount and used injections more commonly, as compared to nonrelapsed group. Longer duration of abuse was also a significant risk factor. Patients with past attempt of opioid detoxification and family history (parental or first degree) of alcohol abuse had decreased possibility of maintaining remission during 1-year follow-up. Relapsed patients were found to abuse their spouse or parents.* Conclusion*. Our study compared profiles of relapsed and nonrelapsed patients after inpatient detoxification and concluded predictors of relapse during 1-year follow-up period. Early identification of predictors of relapse and hence high risk patients might be helpful in designing more effective and focused treatment plan.

## 1. Introduction

Relapse rate after opioid detoxification ranges from 72 to 88% after 12–36 months, despite multidisciplinary endeavors, though a six-month controlled study has shown lower relapse rate (32–70%) [1, 2]. Improvement in this rate can be done by a better understanding of pretreatment risk factors, including patient characteristics, associated with relapse after inpatient detoxification.

Early relapse after inpatient detoxification has been found to be significantly predicted by younger age, greater heroin use prior to treatment, history of injecting, and failure to enter aftercare [3]. Similarly, categories of relapse precipitant have been identified as cognition, mood, external, withdrawal, interpersonal, leaving a protected environment, drug availability, drug-related cues, craving, priming, and social pressure, in a follow-up study of 78 opiates abusers after successful opioid detoxification [4]. Abstinence has also been found to be significantly associated with completion of the 6-week inpatient treatment program and attendance at outpatient aftercare and negatively associated with a family history of substance misuse [5].

In an outpatient detoxification program, interpersonal factors, drug-related cues such as regularly meeting other drug users and being offered drugs, and persistent negative mood states have been found to be associated with relapse into opiate use [6]. Recent developments in cognitive neuroscience point to neurocognitive measures (i.e., brain-imaging measures during cognitive-task performance) as potential predictors of relapse over and above the information gained from self-report measures such as craving [7].

However, in contrast to findings in a long term study, same author earlier studied medium term follow-up outcomes after inpatient treatment of opioid dependence and concluded that patient preadmission characteristics account for a very small proportion of the variance in outcomes [3, 5]. Another 2.5-year follow-up study also found preadmission client characteristics unsuccessful in predicting achievement of abstinence [8].

We studied the possibility that predetoxification patient characteristics might predict relapse at follow-up and thus conducted this 1-year follow-up study to assess the predictors of relapse after inpatient opioid detoxification.

## 2. Materials and Methods

We conducted this study in Department of Psychiatry (Deaddiction unit), Shri Guru Ram Das Institute of Medical Sciences and Research, Vallah, Amritsar, Punjab, India, over two-year time period (1 Jan 2014 to 31 Dec 2015), after permission from Institutional Ethics Committee. Inclusion criteria was any patient with a diagnosis of opioid dependence (as per ICD-10 criteria), admitted for detoxification in the deaddiction unit from 1 Jan 2014 to 31 Dec 2014 and consenting to participate in study. Exclusion criteria included refusal to consent, comorbid other drug addictions (except tobacco), comorbid other psychiatric or significant medical ailments, age <18 years, and known history of any adverse reaction with Naltrexone.

A total of 581 patients were admitted from 1 Jan 2014 to 31 Dec 2014 out of which 115 subjects met defined exclusion criteria or did not meet inclusion criteria. Remaining 466 patients were considered for study. A detailed history was taken and sociodemographic performa (Appendix) was completed for every patient. Average stay of subjects for detoxification varied from two to four weeks depending on withdrawal signs and symptoms. Inpatient detoxification was done as per standard protocol and medications were gradually tapered off to stop after 1–3 weeks except Quetiapine. Tab Quetiapine was used for affective symptoms as per need. After being abstinent from opioids for a minimum of 5–7 days, all patients were discharged on Tab Naltrexone 50 mg o.d. with or without Tab Quetiapine 50–200 mg/d, with regular weekly visits in outpatient unit, for next 1 year.

At least one attendant/caregiver was identified for every patient during inpatient stay, which was mostly a close family member and would stay along with patient. They were made responsible for supervising daily medication at home and were advised to make note if they suspect their patient for any substance abuse. Urine for drug abuse was done randomly to monitor relapse. A total of 2512 random samples were taken, out of which 103 were positive for opioids and they were considered as relapse. None of the patients were positive for any other substance abuse (except tobacco). Patients and their attendants were interviewed regarding relapse, which was defined as abuse of any substance, except tobacco. Alcohol abuse was also not reported by any patient or his attendant.

Adherence therapy of all patients was done at every visit by trained psychologist. Relapsed patients were compared with nonrelapsed patients with respect to their sociodemographic variables as per performa. Patients, who were lost to follow-up, were considered as relapse. Their last observations were carried forward to calculate the final data, rather than considering only the completed subjects, to avoid the bias. We tried to contact them telephonically to ask about their reason for loss to follow-up.

Relapsed and nonrelapsed groups were compared across the variables using chi-square test. A multivariate logistic regression analysis was conducted to identify variables that were independently associated with opiate abstinence. All the tests were two-tailed, and a value of *P* < 0.05 was considered statistically significant.

## 3. Results and Discussion

### 3.1. Sociodemographic Profile

A total of 466 patients were included in our study during the study period (1 Jan 2014 to 31 Dec 2014) and followed up for next 1 year (till Dec 2015). All patients were male. Sociodemographic profile has been provided in Table 1. Majority was in the age range 20–40 and had rural background. Most were married and employed with low income range. Education level was predominantly above matriculation. No significant difference was found between relapsed and nonrelapsed patients.

### 3.2. Drug Use Profile

Heroin was the most common substance of abuse in both groups as evident in Table 2. However, relapsed patients abused greater amount and used injections more commonly, as compared to nonrelapsed group. Longer duration of abuse was also a significant risk factor. Presence of craving at discharge from hospital after detoxification was significant in both groups and logistic regression showed that craving at discharge was significantly associated with relapse (*β* = 6.86, *P* = 0.01). Patients with past attempt of opioid detoxification and family history (parental or first degree) of alcohol abuse had decreased possibility of maintaining remission during 1-year follow-up.

### 3.3. Forensic History Profile

As shown in Table 3, patients with history of police case and incarceration were found to be significantly associated with high risk of relapse. Relapsed patients were also found to verbally and physically abuse their spouse or parents. History of self-harm was not found to be a significant risk factor for relapse.

## 4. Conclusion

(1) Our study compared profiles of relapsed and nonrelapsed patients after inpatient detoxification and concluded predictors of relapse during 1-year follow-up period. High number of young males in both groups represent pattern of drug abuse in population, in general. No female getting admitted for detoxification in our study might be due to high stigma associated with substance abuse; however females have been reported in a significant number depending on city and geographical location, in other studies [9]. Young age has been found to have high risk of relapse after inpatient detoxification [3, 10] but our study did not show any relation of age with relapse. High rural percentage in whole sample shows local area representation, as our tertiary care institute is located in rural area and mostly caters surrounding rural population. A majority of patients in both groups were married and employed, which envisages need to involve family members in treatment process. However, low economic status of patients despite high cost of substances of abuse may suggest downward economic drift of substance abusers as well as alternate source of money being used for buying substances. Education does not appear to act as a deterrent factor for substance abuse or preventing relapse; however economic literacy awareness might be more helpful. Our findings were similar to another study done in a tertiary centre in India, to assess predictors for treatment retention in a tertiary centre in India, where all subjects were males and majority of the sample was married, educated up to matriculation, and employed and belonged to the nuclear family and urban background. Higher socioeconomic status and having a family member with substance use were associated with higher chances of treatment retention [11].

(2) Greater amount of heroin use, longer duration, history of injecting, and >3 lifetime heroin-quit attempts have been found to be significant predictors of relapse [3, 12]. Our study corroborates all these findings. We also found that heroin was the most preferred opioid of abuse among all patients, which might be due to its easy availability. Our patient base represents mainly Amritsar District, which due to its geographical location, being on border with neighboring country Pakistan, is more prone to be affected with smuggled heroin. Further, more than 1 gm of heroin, injection use, and more than 3 years of use prior to detoxification were significantly associated with relapse within 1 year. Craving was found to be present in majority of patients at time of discharge after detoxification, but it did not predict relapse in our study, though compulsive use immediately prior to hospitalization has been found to be significant in another study [13]. We noted that 2 or more previous attempts for detoxification were significantly associated with relapse; however previous studies have shown both supportive and contradictory association [14, 15]. Further, past psychiatry history or admission (except substance abuse) was not found to be associated with relapse in our findings. We also noted significant history of alcohol abuse in parents and first-degree relatives of relapsed patients, similar to another follow-up study in which 19% patients reported history of parental alcohol misuse, but surprisingly opioid abuse history in family was not found to be associated with relapse in our study [5]. Inpatient treatment and regular follow-ups have also been found to be significantly associated with abstinence [3, 5, 16]. Our studies design ensured admission and regular follow-ups for all patients which might have been a reason for lower overall relapse rate as compared to other researches.

(3) We found police case and imprisonment history to be significant in relapsed cases as shown in Table 2. One reason of high relapse in these patients could be presence of antisocial traits which itself is a risk factor for substance abuse. However, client characteristics were not found to be associated with relapse in two different studies, one a 2.5-year follow-up study and the other was a medium term follow-up study [5, 8]. One of the reasons for different results could be that, in 2.5-year follow-up study, analysis was done 2.5 years after detoxification. Another reason could be difference in culture and treatment setup. In our culture, patients without antisocial traits are better monitored by their family members while those with such traits may not be very compliant to their family values.

Another reason for high relapse in the patients in our study could be habituation of offenses. Illegality is a major deterrent for opioid abuse but involvement in police cases and being in jail possibly habituates the offender, who finally does not find it as a deterrent anymore. Incarceration was found to be most common cause of dropout in a community based Indian study [9]. Involvement in legal issues has been reported as one of the major factors for treatment failure [17]. Our study also found history of verbal and physical abuse of spouse or parents as a significant risk factor for relapse. Positive family functioning and relationships have been reported to be significantly associated with improvement at follow-up [18, 19]. Problem with spouse was found to be a precipitant factor in another follow-up study [20].

To conclude, early identification of predictors of relapse and hence high risk patients might be helpful in designing more effective and focused treatment plan. More research is needed to explore patient characteristics based on our study which may help to decrease recurrent admissions and hospital expenses.

## 5. Limitations

Relapse criteria relied on interview with patients and their accompanying attendants, rather than regular urine screening tests, although random tests were done.

## Figures and Tables

**Table 1:** Sociodemographic profile.

	Relapsed (*n* = 147)	Nonrelapsed (*n* = 319)	Chi-square value	Signific.
*Age*				
Below 20 years	45 (30.61%)	70 (21.94%)	4.70	Ns
20–40 years	80 (54.42%)	195 (61.13%)		
Above 40	22 (14.50%)	54 (16.93%)		

*Resid. status*				
Rural	102 (69.39%)	223 (69.90%)	0.01	Ns
Urban	45 (30.61%)	96 (30.09%)		

*Marital status*				
Single	41 (27.89%)	82 (25.71%)	0.25	Ns
Married	85 (57.82%)	190 (59.56%)		
Divor./separated	21 (14.29%)	47 (14.73%)		

*Empl. status*				
Employed	61 (41.50%)	148 (46.39%)	3.20	Ns
Unemployed	56 (38.10%)	95 (29.78%)		
Prev. employed	30 (20.40%)	76 (23.82%)		

*Income (INR pm)*				
0–<10000	77 (52.38%)	165 (51.72%)	0.78	Ns
10000–<20000	37 (25.17%)	91 (28.52%)		
20000–above	33 (22.45%)	63 (19.74%)		

*Education*				
Illiterate	28 (19.05%)	45 (14.11%)	3.06	Ns
Up to matric.	39 (26.53%)	79 (24.76%)		
Above matric.	80 (54.42%)	195 (61.13%)		

**Table 2:** Substance misuse.

	Relapsed (*n* = 147)	Nonrelapsed (*n* = 319)	Chi-square	Level of sign.
*Substance misuse history*				

*Principal opiate of use*				
Morphine	10 (6.80%)	30 (9.40%)	5.15	Ns
Poppy husk	12 (8.16%)	31 (9.71%)		
Heroin/smack	70 (47.61%)	150 (47.02%)		
Tramadol	15 (10.20%)	20 (6.26%)		
Dextropropoxyphene	9 (6.12%)	29 (9.09%)		
Diphenoxylate	3 (2.04%)	9 (2.82%)		
Combination of opiates	28 (19.04%)	50 (15.67%)		

*Quantity (heroin per day)*				
Less than 0.5 gm	23 (17.68%)	110 (34.48%)	57.52	0.01
0.5–1 gm	38 (25.85%)	136 (42.63%)		
More than 1 gm	86 (58.50%)	73 (22.88%)		

*Predominant route*				
Chase/smoke	4 (2.72%)	70 (21.94%)	43.85	0.01
Inject	70 (47.61%)	90 (28.21%)		
Oral	28 (19.04%)	83 (26.01%)		
Multiple	45 (30.61%)	76 (23.82%)		

*Duration of abuse*				
<1 year	10 (6.80%)	101 (31.66%)	48.85	0.01
1–3 years	47 (31.97%)	118 (36.99%)		
>3 years	90 (61.22%)	100 (31.34%)		

*Craving at discharge*				
Yes	123 (83.67%)	248 (77.74%)	2.18	Ns
No	24 (16.32%)	71 (22.25%)		

*Number of previous attempted opiate detoxifications*				
0	45 (30.61%)	101 (31.66%)	49.55	0.01
1	13 (8.84%)	118 (36.99%)		
Two or more	89 (60.54%)	100 (31.34%)		

*Past (nonaddiction) psychiatric history*				
Yes	36 (24.48%)	102 (31.975)	2.71	Ns
No	111 (75.51%)	217 (68.02%)		

*Inpatient psy treatment*				
Yes	9 (6.12%)	21 (6.58%)	0.03	Ns
No	138 (93.87%)	298 (93.41%)		

*Parental alcohol misuse*				
Yes	88 (59.86%)	57 (17.86%)	82.80	0.01
No	59 (40.13%)	262 (82.13%)		

*Parental opiate abuse*				
Yes	42 (28.57%)	109 (6.80%)	1.44	Ns
No	105 (71.42%)	210 (65.83%)		

*First-degree relative alcohol abuse*				
Yes	95 (64.62%)	74 (23.19%)	74.72	0.01
No	52 (35.37%)	245 (76.80%)		

*First-degree relative opiate abuse*				
Yes	55 (37.41%)	125 (39.18%)	0.13	Ns
No	92 (62.58%)	194 (60.47%)		

**Table 3:** Forensic history.

	Relapsed (*n* = 147)	Nonrelapsed (*n* = 319)	Chi-square	Level of sign.
*Forensic history*				

*Police case registered*				
Yes	24 (16.32%)	18 (5.64%)	14.01	0.01
No	123 (83.67%)	301 (94.35%)		

*Imprisonment*				
Yes	12 (8.16%)	7 (2.19%)	9.17	0.01
No	135 (91.83%)	312 (97.81%)		

*History of verbal abuse*				
Yes	100 (68.02%)	111 (34.80%)	44.85	0.01
No	47 (31.97%)	208 (65.20%)		

*History of physical abuse*				
Yes	65 (44.22%)	47 (14.73%)	47.91	0.01
No	82 (55.78%)	272 (85.27%)		

*History of self-harm*				
Yes	8 (5.44%)	20 (6.27%)	0.12	Ns
No	139 (94.55%)	299 (93.73%)		

## Appendix

### Performa for Research Study

Name:
Sex:
MRD No.:
Serial No.:

*Socio-Demographic Profile*

Age
  Below 20 years
  20–40 years
  Above 40
Resid. Status
  Rural
  Urban
Marital Status
  Single
  Married
  Divor./Separated
Empl. Status
  Employed
  Unemployed
  Prev. employed
Income (INR pm)
  0–<10000
  10000–<20000
  20000–Above
Education
  Illiterate
  Up to Matric.
  Above Matric.

*Substance Misuse*

Principal opiate of use
  Morphine
  Poppy Husk
  Heroin/ Smack
  Tramadol
  Dextropropoxyphene
  Diphenoxylate
  Combination of opiates
Quantity (heroin per day)
  Less than 0.5 gm
  0.5–1 gm
  More than 1 gm
Predominant route
  Chase/smoke
  Inject
  Oral
  Multiple
Duration of abuse
  <1 year
  1–3 years
  >3 years
Craving at Discharge
  Yes
  No
No. of previous attempted opiate detoxification
  0
  1
  Two or more
Past (non-addiction) Psychiatric history
  Yes
  No
In-patient psy treatment
  Yes
  No
Parental Alcohol misuse
  Yes
  No
Parental opiate abuse
  Yes
  No
First rel. alcohol abuse
  Yes
  No
First relative opiate abuse
  Yes
  No

*Forensic History*

Police Case Registered
  Yes
  No
Imprisonment
  Yes
  No
History of verbal abuse
  Yes
  No
History of physical abuse
  Yes
  No
History of Self Harm
  Yes
  No
